# Experimental and Numerical Study of Steel–Concrete Composite Beams Strengthened under Load

**DOI:** 10.3390/ma17184510

**Published:** 2024-09-13

**Authors:** Piotr Szewczyk

**Affiliations:** Faculty of Civil and Environmental Engineering, West Pomeranian University of Technology in Szczecin, al. Piastów 17, 70-310 Szczecin, Poland; szewczyk@zut.edu.pl

**Keywords:** steel–concrete composite beams, strengthening, FEM, welding

## Abstract

This study analysed the strengthening process of a classical steel–concrete composite beam. The beam consisted of a reinforced concrete slab connected by shear studs to an IPE steel profile. The key idea was that the composite beam was strengthened under load. This process simulated an actual reinforced structure that is always subjected to dead loads, with possible service loads. This study assumed that strengthening was implemented to increase the load-carrying capacity and stiffness, not as a way for simulation a repair. The strengthening consisted of expanding the steel part of the beam by welding an additional plate to the bottom flange of the IPE profile. This study included the results of numerical analyses conducted in Abaqus software and lab results. A three-dimensional numerical model was created, taking into account the non-linear behaviour of concrete and steel, the susceptibility of the composite at the joint plane, and the residual stresses created during welding. A full-scale strengthening of the composite beams under load was carried out. Comparison of the results obtained in the experimental tests and numerical analyses showed a very high convergence of the results, as well as in terms of the non-linear operation of steel and concrete. This confirmed the validity of the created numerical model, which can be the basis for further research into the process of optimal strengthening of composite elements.

## 1. Introduction

Composite components, i.e., made up of at least two materials that differ in their properties, are used in many industries. The ability to optimise the design of these components in order to make the best use of both materials’ properties makes composite elements a popular construction choice. As a result, it is possible to obtain elements with reduced weight at lower labour and cost. The most popular solution, which has been used for years in engineering, is the use of steel–concrete composites as beams or columns. Similarly, aluminium–concrete [1,2,3,4], aluminium–timber [5,6,7,8], steel–timber [9,10,11,12] or timber–concrete [13,14,15,16] composites can be found, particularly in historic buildings. Non-obvious combinations such as those described in [17,18] are also emerging. With the development of new building materials, such as LVL wood, high-strength concrete or lightweight concrete, and new composite techniques, continuous research on these structures is still needed. As structures made with these composites are being built, the need for renovations and reinforcements will also become increasingly necessary. Reinforcing an existing structure requires higher expertise than designing a new one. The level of difficulty is further increased by having to analyse a composite element composed of materials with different properties.

The need to reinforce structures can emerge from a number of factors. It may be a result of an emergency related to the failure of structural elements, e.g., as a result of design or execution errors, improper operation of the facility or random events that are difficult to anticipate. On the other hand, the need for reinforcement may arise from the need to increase the load-bearing capacity or stiffness of the structural elements due to renovation, redevelopment or change of use. The increasing adaptation of existing facilities to a new role is the result of a limited number of vacant plots in city centres, the introduction of sustainable development principles and the reduction of emissions associated with the production of building materials. Consequently, these problems will frequently be faced by civil engineers. This study focused on the former, simulating the need to adapt a building to a new role.

Increasing the load-bearing capacity and stiffness of a structure can be performed in a number of ways. An obvious way to improve the load-bearing capacity is to increase the cross-sectional area [19,20,21,22]. However, for elements prone to loss of stability, e.g., compressed or bended elements, this may not be sufficient. In such cases, it important to reduce the buckling length or to block the possibility of local buckling [23,24,25,26,27,28]. The desired effect may also be achieved by, e.g., modifying the static scheme, prestressing the structure using tendons [29,30,31] or adjusting the stresses by changing the support position of the statically non-determinable structure.

The effectiveness of the strengthening process of steel elements is strongly influenced by the level of allowable stresses. There are two options: leaving the elements being reinforced and strengthening them in the elastic range, or allowing partial yielding. Remaining in the elastic range assumes that the strengthening element only carries the load applied after reinforcement is installed, which is an inefficient solution. In this case, the increase in load capacity depends on the level of stress in the reinforced element. Only a plastic analysis allows the stresses to be redistributed and the newly incorporated material to be fully utilised. In this case, the stress level in the reinforced element does not affect the ultimate load capacity of the section. Such an analysis was carried out in the research presented in this article.

The built-in material determines the technical feasibility of strengthening solutions. Steel constructions, which can be welded, offer a great spectrum of methods. However, residual welding stresses and deformations must be taken into account, as they will affect the final dimensions of the reinforced structure. Welding components under load is a complicated task [32,33,34,35]. Weld distortion is dependent on the stress level at the weld site. In addition, the behaviour of the tension and compression components during welding is completely different. Generally, as tensile stress increases, the value of weld shrinkage decreases, while the opposite occurs during compression. However, this is not a linear relationship. When strengthening reinforced concrete structures, it is important to remember concrete’s brittle behaviour. In this situation, it is important to ensure that the new concrete layer is properly bonded to the existing one and that there will be stresses associated with shrinkage development over time.

In this study, an attempt was made to measure and evaluate the entire process of strengthening a steel–concrete composite beam, which can be found in both steel and reinforced concrete structures. A three-dimensional non-linear FEM numerical model was prepared that included the whole strengthening process. The model was validated on the basis of laboratory tests on a large technical scale. A key assumption in the whole analysis was that the element was strengthened under load. The research was carried out in the destructive range, which made it possible to trace the plastic redistribution of stresses in the reinforced and reinforcing elements. In addition, the strengthening plate was welded into the beam, which required an estimation of the deformations in the element under load. The value of welding deformation depends on the value and nature (compression or tension) of the stresses at the welding site. This is an issue that has not been systemically solved and requires further research.

## 2. Materials and Methods

### 2.1. Test Element Design

**Figure 1:**
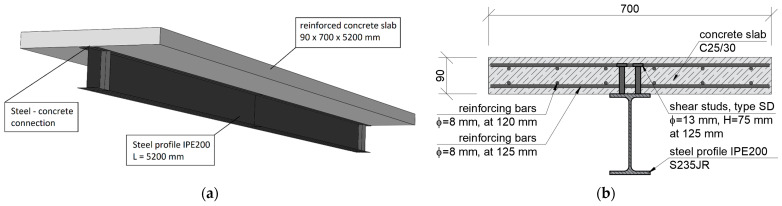
Steel–concrete composite beam: (**a**) overview and (**b**) cross-section.

This study was performed on a steel–concrete composite beam (Figure 1a). It was investigated as a bended free-supported beam. With this scheme of operation, it was possible to make full use of the embedded materials in the structure. The cross-section was designed so that the neutral axis of the beam was as close as possible to the joint plane. The lower, tensile part of the beam is S235JR steel with an IPE200 rolled profile. The upper, compressed part was formed by a 700 × 90 mm reinforced concrete slab. The slab was made with C25/30 concrete reinforced with 8 mm Bst500s steel bars. The joint was made by installing type SD shear studs (Köco Köster & Co., Ennepetal, Germany), which were 75 mm in height and 13 mm in diameter. The connectors were used in pairs, spaced at 125 mm. The cross-section of the beam is shown in Figure 1b. The total length of the beam was 5200 mm and was chosen to ensure safe support spacing of 5000 mm. Additional 10 mm stiffening plates were designed at the support locations.

Two types of models were prepared, a numerical one and experimental ones, which are described in Section 2.2 and Section 2.3, respectively.

The adopted beam geometry, with the reinforced concrete slab connected to the top flange of the steel beam, results in a lack of lateral–torsional buckling. The strengthening solution was created by increasing the cross-sectional area of the element. It is possible to extend the steel section by welding an additional plate or rolled profile, while the reinforced concrete section can be increased by adding an additional layer of concrete. This study focused on the first solution, the expansion of the steel part. Reinforcing the reinforced concrete section will be the focus of further research.

### 2.2. Numerical Model

**Figure 2:**
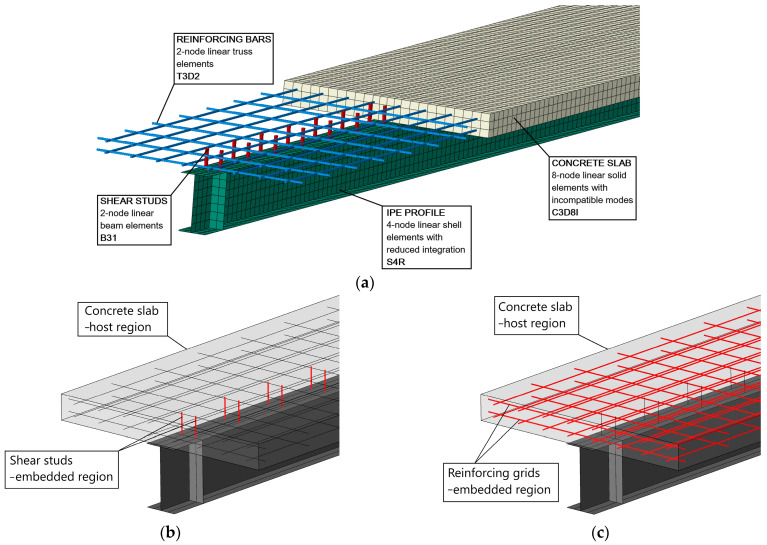
FEM model of steel–concrete composite beam: (**a**) general view, (**b**) details of the steel–concrete joint, and (**c**) reinforcement details in the concrete slab.

A three-dimensional FEM numerical model of the analysed beams was created in Abaqus/CAE 2016 software (Figure 2). The software has very significant potential for conducting non-linear analyses, especially with consideration of the complex numerical models of the materials used, such as a brittle model of concrete (CDP model described in Section 2.5.2). The reinforced concrete slab was modelled using linear solid elements with incompressible modes C3D8I. The steel beam was modelled with shell elements with reduced integration S4R.

Proper modelling of the joint plane was crucial in this case. It was decided to model the exact joint mechanism. Linear beam elements B31 were added to the top flange of the IPE200 steel beam with spacing and dimensions consistent with the shear studs’ spacing. These elements are presented in Figure 2b in red colour. Their interaction with the reinforced concrete slab was then ensured. The shear studs became an embedded region in the concrete slab that had become a host region for them. The rebars in the reinforced concrete slab was modelled similarly. In Figure 2c, two reinforcement grids (red) made of linear truss elements T3D2 were embedded in the slab. A hard contact was assumed at the interface between the slab and the beam with a friction coefficient adopted. The numerical model takes into account the non-linear behaviour of the materials: the ductility of steel and the brittleness of concrete, as described in detail below.

### 2.3. Experimental Specimens

**Figure 3:**
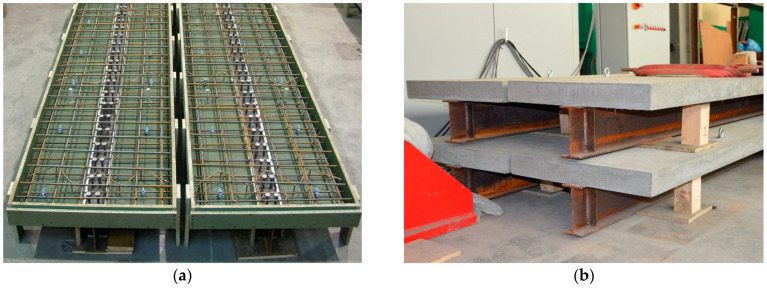
Composite beams: (**a**) beams before concreting with visible reinforcement and shear studs, and (**b**) produced beams.

Four identical composite beams were prepared. Figure 3a shows the beams just before concreting. A separate formwork was prepared for each beam, in which a steel I-beam with welded connectors and two reinforcement grids were placed. Figure 3b presents four composite beams ready for testing. One of the beams at the preparation stage was damaged. This article presents the results of the three remaining beams. The material samples were taken to determine the mechanical properties of steel and concrete.

### 2.4. Steel

#### 2.4.1. Material Tests

**Figure 4:**
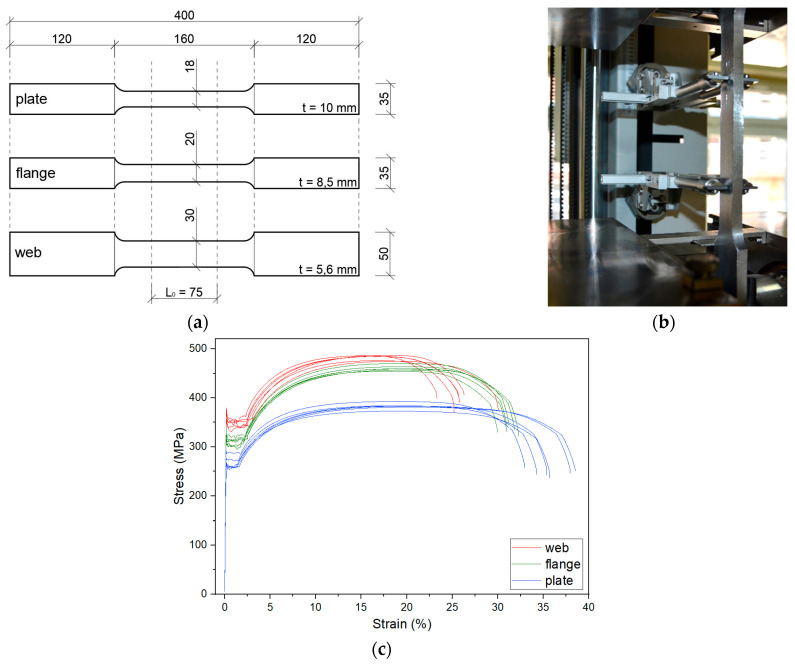
Static tensile strength test: (**a**) sample dimensions, (**b**) sample in the apparatus, and (**c**) stress–strain curves.

The steel samples were taken from the IPE200 profile (separately from the flange and web) and from the strengthening plates (Figure 4a). These three groups of samples had a different thickness *t*. The same original gauge length *L_o_* = 75 mm was common to all samples. Six specimens were prepared from each part of the beam. A static tensile test was carried out in accordance with ISO standard [36] (Figure 4b), from which the upper (*R_eh_*) and lower yield strengths (*R_el_*), tensile strength (*R_m_*), Young’s modulus (*E*) and maximum elongation at break (*A*) were determined. The results obtained in the form of mean $\bar{x}$ and standard deviation *s* for 3 test series are shown in Table 1, and in Figure 4c as a stress–strain diagram.

#### 2.4.2. Material Model

**Figure 5:**
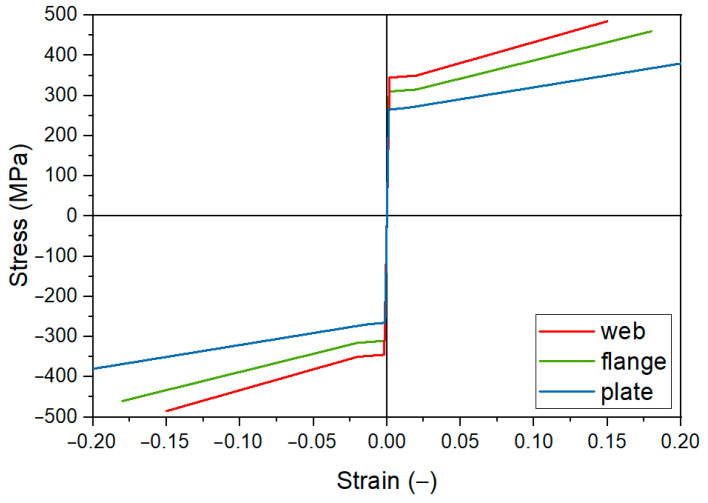
Non-linear numerical model of steel.

Based on the determined characteristics, a non-linear numerical model of steel was created in Abaqus/CAE software. The steel was modelled as an elastic–plastic material with yield point and strain hardening (three-linear model). Independent models were developed for the flange and web of the IPE200 beam and the reinforcing plate as shown in Figure 5.

### 2.5. Concrete

#### 2.5.1. Material Tests

**Figure 6:**
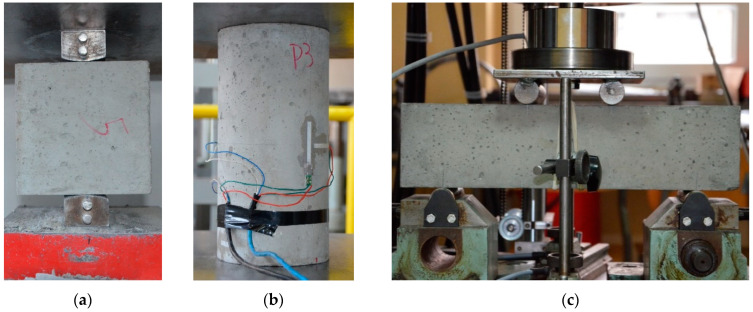
Concrete samples: (**a**) 150 mm cubes, (**b**) 300 × 150 mm cylinders, and (**c**) 100 × 100 × 400 mm beams.

Concrete 150 × 150 × 150 mm cubes (Figure 6a), 300 × 150 mm cylinders (Figure 6b) and 100 × 100 × 400 mm prisms (Figure 6c) were cast during the preparation of the slab. The specimens were used to determine the compressive and tensile strengths. The results are presented in Table 2. Using the strain gauges shown in Figure 6b, Young’s modulus *E* = 32 GPa and Poisson′s ratio *ν* = 0.18 were also determined.

**Figure 7:**
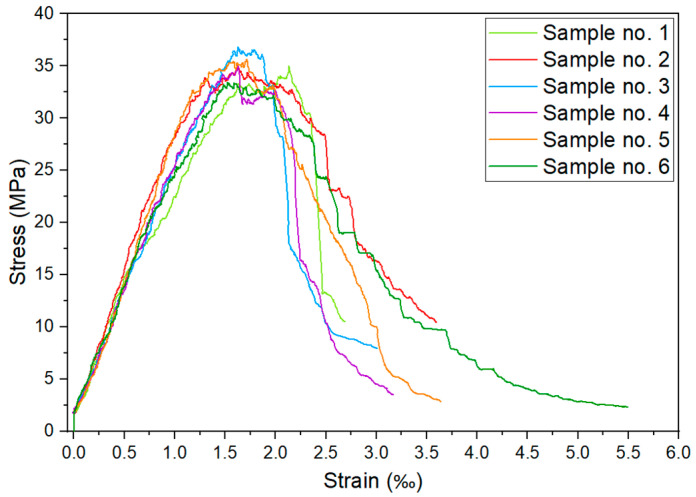
Residual compressive strength of specimens.

Additional tests were performed to prepare the numerical model. Residual compressive strength was determined, as shown in Figure 7. This was used to determine the parameters related to the degradation of concrete stiffness.

#### 2.5.2. Material Model

**Figure 8:**
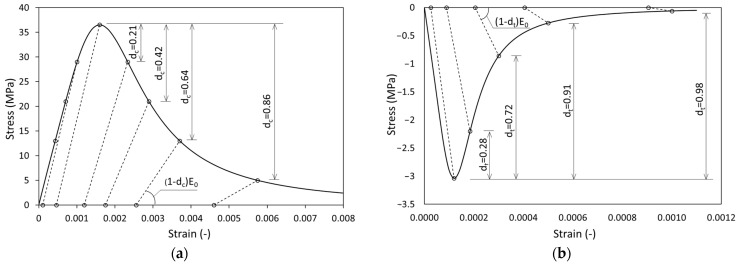
CDP model: (**a**) range of compression stresses and (**b**) range of tension stresses.

The properties of concrete were recreated using the Concrete Damage Plasticity (CDP) model [37,38,39,40,41]. This model has been successfully used for numerical simulations of structures made of concrete and other brittle materials [42,43,44,45,46,47]. CDP allows the independent modelling of the behaviour of concrete in compression (Figure 8a) and tension (Figure 8b). It also allows the inclusion of the stiffness degradation parameter *d*, independently in the analysis of compression *d_c_* and tension *d_t_*. Due to the widely available theoretical basis of the CDP model and the popularity of using it in FEM simulations, details of this model are not included in this paper.

### 2.6. Initial Assumptions

The laboratory tests were performed to reflect as close as possible the behaviour of the actual construction. The most important assumptions for achieving this are outlined below.

#### 2.6.1. Cyclic Preload

**Figure 9:**
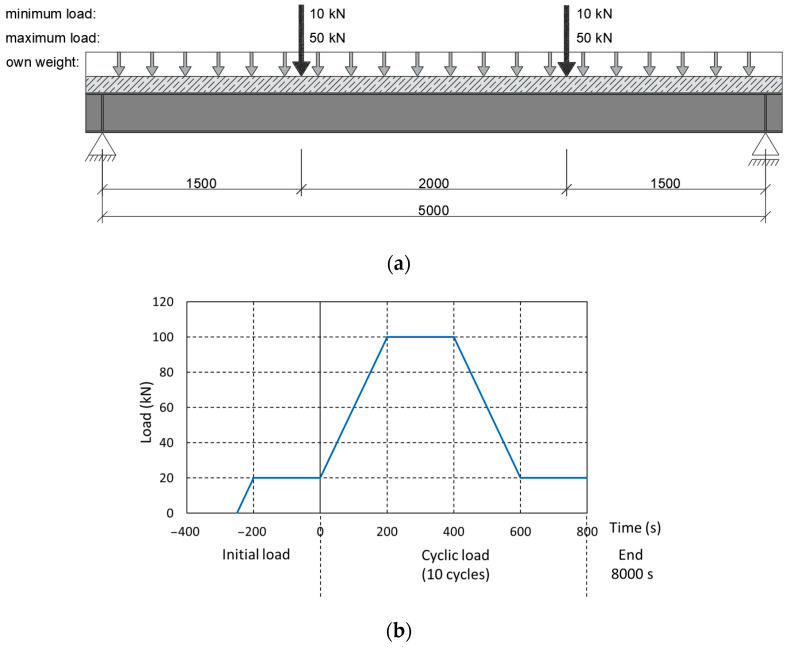
Cyclic preload: (**a**) static scheme of the initial cyclic load and (**b**) cyclical load program.

Every structure behaves slightly differently during the initial loading cycles compared to subsequent periods of operation. During the initial loads, any occurring play in the joints is eliminated and the mechanical properties of the materials, e.g., the Young’s modulus of the concrete, are stabilised, as can be seen, for example, when measuring the displacements of the structure. Therefore, prior to the main tests, the beams were subjected to a series of preloads simulating changes in the imposed loads over time. The loading scheme is presented in Figure 9a. The same scheme was then used for the main tests.

Each beam was subjected to a cycle of static loading consisting of four stages (Figure 9b):

- Ramp up to 100 kN in 200 s (speed—0.4 kN/s);
- Maintain the load for 200 s;
- Ramp down to 20 kN in 200 s (speed—0.4 kN/s);
- Maintain the load for 200 s.

The tests were carried out until the results stabilised in successive measurement cycles, which occurred after about 10 cycles. The complete test took about 2.5 h.

Table 3 presents the values of the loads applied during the test and the corresponding stresses. A dead load of the beam and auxiliary elements (DL) of 13 kN was added to the load value. The maximum stresses in the bottom flange of the steel beam were close to the theoretical yield strength. The stresses in the upper part of the reinforced concrete slab were slightly below the calculated compressive strength. This stress level may correspond to the real-life value in a structure.

**Figure 10:**
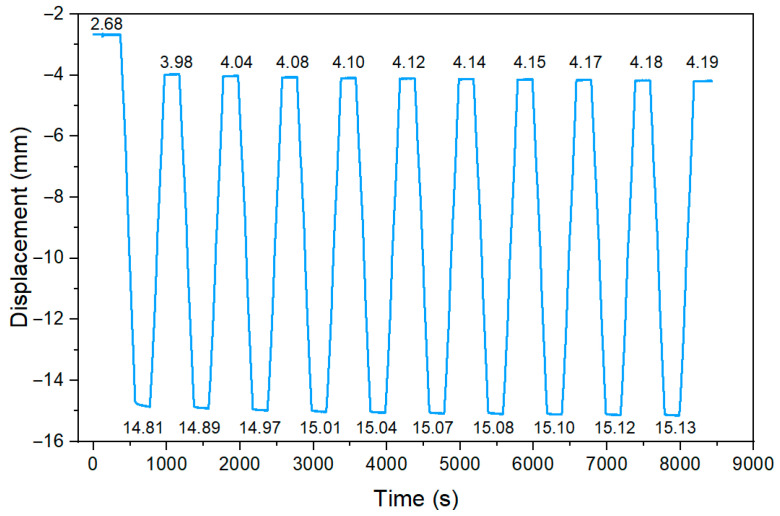
Increase in deflection in subsequent cycles.

Figure 10 shows an example of a vertical displacement measurement at the centre of the span of one of the tested beams. For each test cycle, the average value of the displacement at the holding stage was measured. Significant increase in deflection was observed after the initial cycle. The deflection after the first unloading from 100 kN to 20 kN increased by 1.3 mm. In subsequent loading cycles, the increments of displacement became increasingly smaller until the results stabilised at a constant level. The beam behaviour clearly shows the effect of the applied load cycles on the behaviour of the structure.

#### 2.6.2. Strengthening under Load

The second part of the study focused on the stresses in the beam during strengthening. Existing structures always have a certain level of stresses, which are caused, e.g., by the dead load itself. The structure is subjected to permanent or service loads. It is possible to decrease the load during strengthening, e.g., by using scaffolding with hydraulic cylinders. This generates additional costs and requires the space beneath to be taken out of service for a longer period of time. Therefore, this work assumed that the structure would be subjected to a load during strengthening, and the value of this load would be one of the variables of the parametric analyses [48,49].

One of the most important questions is whether the level of stresses during strengthening affects the effectiveness of the process as a whole. The result depends on the adopted criterion of allowable stresses. The first possibility is to remain in the elastic range of deformation. With this assumption, the strengthening element carries only the load applied after installation, while the reinforced element carries the load applied before as well as after strengthening. This method is clearly inefficient for strengthening under load. Alternatively, if at least partial yielding of the reinforced element is allowed, there is a favourable phenomenon of plastic stress redistribution between the reinforcing and reinforced elements. In this way, the reinforcing part can be incorporated to carry the loads applied to the structure before reinforcement. From the point of view of the load-bearing capacity, there is no need to unload the structure, since the ultimate capacity of the section is independent of the stress level at which the strengthening took place. From an economic point of view, the application of the second criterion is clearly preferable. It leads to more rational use of the newly installed material and does not require reducing the stresses during strengthening. This approach was used in this study.

**Figure 11:**
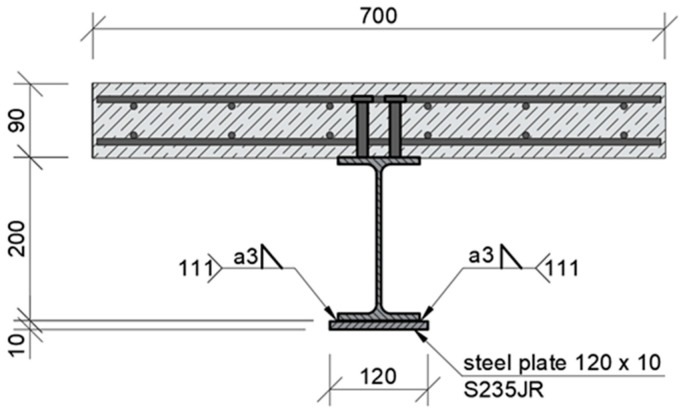
Location of the strengthening plate.

The inclusion of initial stresses during strengthening, both in the experimental studies and numerical analyses, required the whole process to be divided into three main parts: before strengthening, during strengthening, and after amplification:

1. The first step was the introduction of a preload, simulating the stress state of the structure before strengthening. This load was set in such a way that the ultimate limit state condition was maintained during each strengthening step. The welding of the structure, through rapid heating to significant temperatures, caused local weakening of the section. This can be taken into account in the calculation by assuming a reduced cross-sectional area. A force of 60 kN was taken as the preload. Together with the dead load, the assumed level of the preload results in 163 MPa of stress in the bottom flange of the steel beam (welding point) (Figure 11). After taking into account the reduction in stiffness during welding, the stresses increase to 228 MPa, which constitutes 97% of the allowable stress.
2. The second stage was the strengthening process, which consisted of welding an additional plate to the bottom flange of the steel beam. The dimensions of the plate were optimised based on the energy parameters [50]. A plate with a section of 10 mm × 120 mm and a length of 3300 mm was adopted. Its location in the beam cross-section is shown in Figure 11. The strengthening plate was 20 mm wider than the bottom flange of the IPE200 beam to improve the quality of the fillet weld at the PB position.
3. Once the strengthening process was complete and the structure had cooled completely, the load was increased until the yielding and failure of the models. This approach allowed the recording of the plastic load-bearing capacity of the beam resulting from the full redistribution of stresses between the reinforced element and the strengthening. The failure mechanism of the beam was also determined.

### 2.7. Numerical Analysis

The process of strengthening the composite beam by welding, as presented in the previous section, was modelled in Abaqus software. As in the experimental part, the whole process was divided into several stages.

The first stage simulated the stress state before strengthening. The stresses only appeared in the reinforced element. The strengthening plate was connected to the beam in the following strengthening stage; thus, it did not operate in this stage of the numerical model.

**Figure 12:**
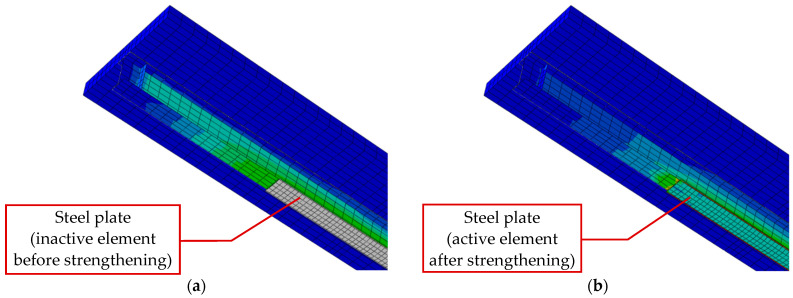
Numerical model with strengthening plate: (**a**) with inactive plate and (**b**) with operating plate.

The presented case is an interesting and non-standard issue in numerical analysis, as it requires the model parameters to be changed during simulation. The strengthening element starts operating in one of the subsequent simulation steps. The plate must incorporate the deformation of the I-beam. Figure 12 shows the same numerical model in different calculation steps. Figure 12a presents the beam before strengthening, where the reinforcing plate is inactive (no result map). Figure 12b presents the beam after strengthening, where the strengthening plate carries the load.

The final step simulated the increase in the imposed load. In both the numerical analysis and experimental tests, the load could be increased up to the failure of the beam, allowing the yielding and stress redistribution between the strengthened and strengthening elements to be monitored.

**Figure 13:**
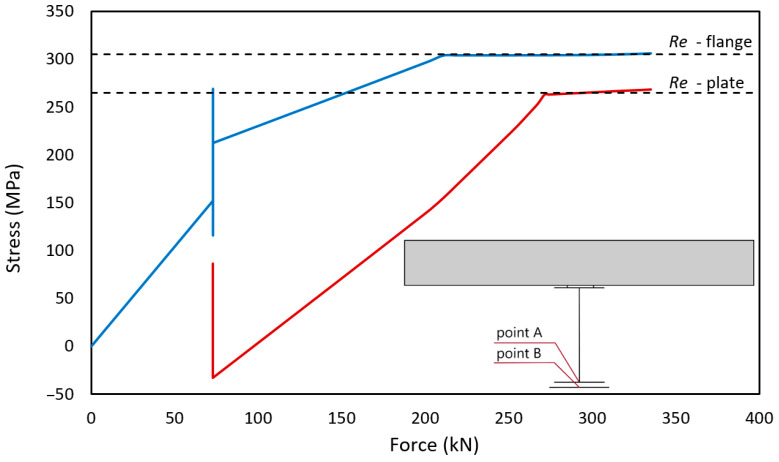
Stress distribution in the strengthened element (point A-blue line) and the strengthening element (point B-red line).

To illustrate yielding and stress redistribution, two adjacent points in the central section of the beam were selected for the numerical analysis. Point A located in the IPE200 bottom flange while point B in the strengthening plate. Figure 13 shows the increase in normal stress at both points as a function of load. Up to a load of 73 kN, stresses occur only in the composite beam. The point corresponding to a load of 73 kN is very interesting; this is the moment of strengthening the beam. Immediately after welding, due to the shrinkage of the longitudinal fillet welds, the reinforcing plate was compressed, thus imposing compressive stresses. A change in stress was also visible in the reinforced beam. This is a beneficial phenomenon, as it increases the possible load in the elastic range. As shown in Figure 13, three distinctive ranges of operation can be observed both for the I-beam and the strengthening plate. The first is the elastic operation of both elements (0–211 kN). The second involves the yielding of the I-beam, but the elastic operation of the strengthening plate (211–271 kN). The third range involves the yielding of both materials (>217 kN). The strengthening plates and I-beams were made from slightly different steel, with varying yield strengths (Re), as marked in the chart.

**Figure 14:**
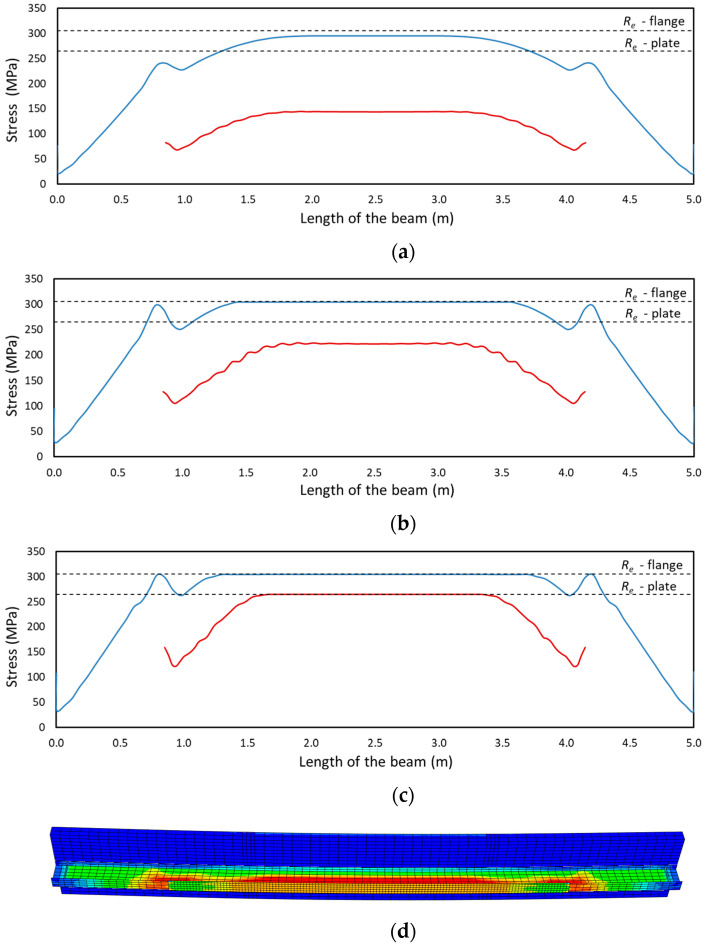
Stress distribution in the bottom flange and strengthening plate: (**a**) at a load of 200 kN, (**b**) at a load of 250 kN, and (**c**) at a load of 280 kN. (**d**) von Mises stress distribution map.

The three stages of operation are presented in Figure 14 a-c as normal stress distributions in the bottom flange of the I-beam (blue line) and in the strengthening plate (red line). Figure 14a presents the results for a load of 200 kN. The bottom flange is already close to yielding, while the stresses in the strengthening plate only reach 150 MPa. The yielding of the I-beam allows additional stresses to develop in the strengthening plate, as shown in Figure 14b (250 kN). A further increase in load results in the yielding of the strengthening plate, as shown in Figure 14c. The stress state shown corresponds to a load of 280 kN. An example of stress distribution in the I-beam and strengthening plate is shown in Figure 14d.

### 2.8. Experimental Tests

Due to the extensive forces applied, the tests required a suitably rigid test bench. The test bench consists of a ribbed steel frame that was 5 m high and 4 m wide. A Zwick/Roell hydraulic actuator was attached to the frame, capable of exerting a load of 600 kN. The actuator allowed the control of both the force and displacement of the piston, which was essential for the tests.

**Figure 15:**
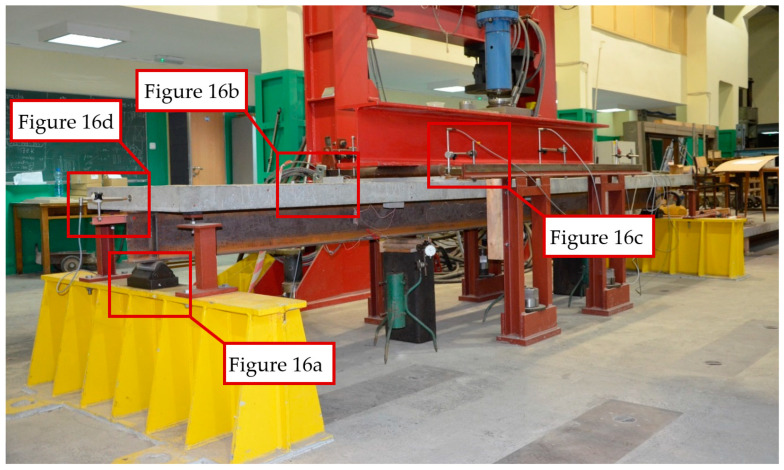
Beam placed on the test bench.

**Figure 16:**
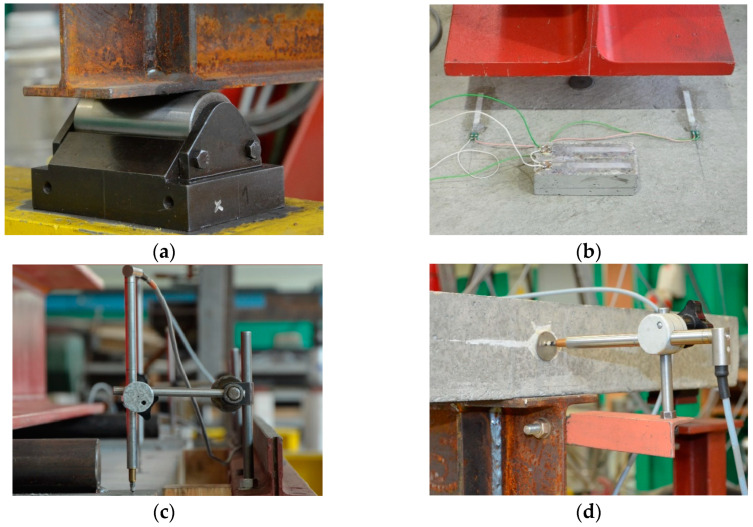
Detailed view of the test bench: (**a**) rolled support, (**b**) strain gauge, (**c**) LVDT transducers for vertical displacement, and (**d**) LVDT transducer for measuring joint susceptibility.

**Figure 17:**
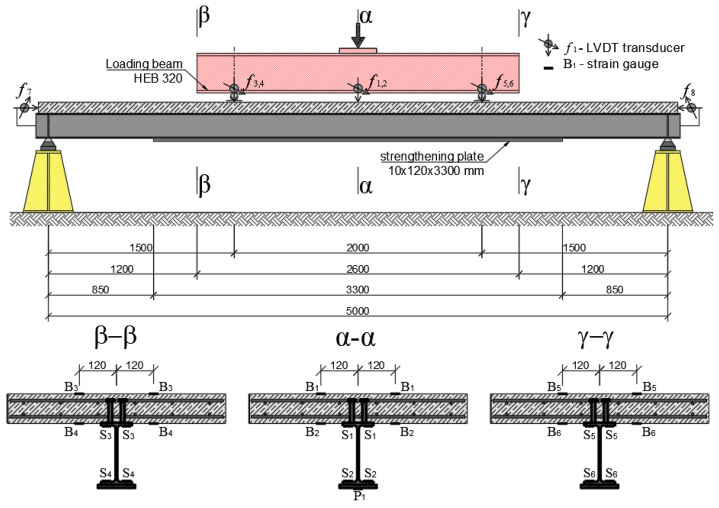
Schematic of the dimensions and location of the measuring sensors on the test bench.

Figure 15 shows one of the beams before the test. The beam was placed on a roller support allowing free rotation (Figure 16a). A number of strain gauges (Figure 16b) and linear variable differential transformer (LVDT) transducers were placed on the beam to measure the horizontal displacement (Figure 16c) and joint susceptibility (Figure 16d). The location and type of all measuring points are presented in Figure 17 and Table 4.

**Figure 18:**
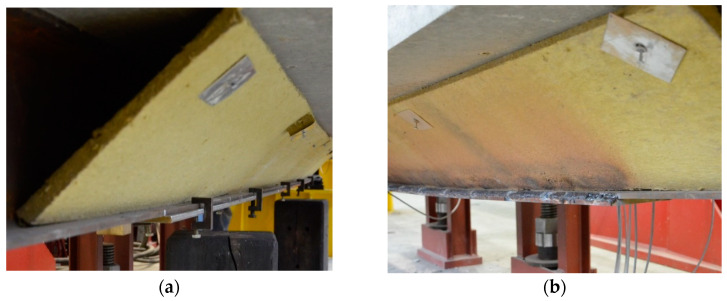
Strengthening plate: (**a**) plate temporarily fixed to bottom flange, and (**b**) plate after welding.

At the start of each test, a preload of 60 kN was introduced. This force was intended to simulate the load on the structure and was kept constant throughout the strengthening process. The strengthening plate was then fixed. To ensure proper adherence to the curvature of the bottom flange of the I-beam, the plate was fixed to the bottom flange using steel ties, as shown in Figure 18a. Welding was carried out using the manual metal arc method (MMA) with rutile-covered electrodes of 3.2 mm in diameter. The location of the welds is presented in Figure 11 and Figure 18b. The length of the welds placed at a time was determined by the length of the electrodes and was approximately 120 mm. The ends of the reinforcing plate were welded first. The welds were then placed starting from the centre of the beam towards the ends. Because the welds were placed on both sides of the beam, the work was carried out by two welders, each equipped with a separate welding machine. The welds were placed separately.

**Figure 19:**
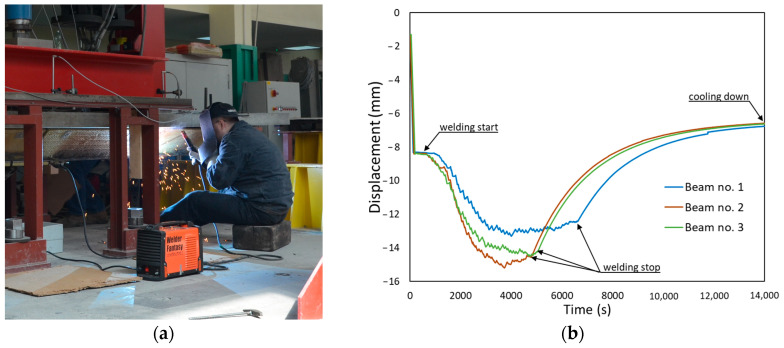
Welding process: (**a**) the MMA process, and (**b**) vertical displacements of the beams during welding.

During welding (Figure 19a), displacement values were measured. The variability in their values over time is shown in Figure 19b. The vertical displacement of the beam increased due to the rapid heating of the lower part of the steel beam. As it cooled down, the opposite trend of displacement appeared due to welding shrinkage. The actuator piston changed its position to allow free deformation, while simultaneously maintaining the load. The process took three to four hours. Precise control of the actuator minimized the force fluctuation during welding to a maximum of 10 N.

**Figure 20:**
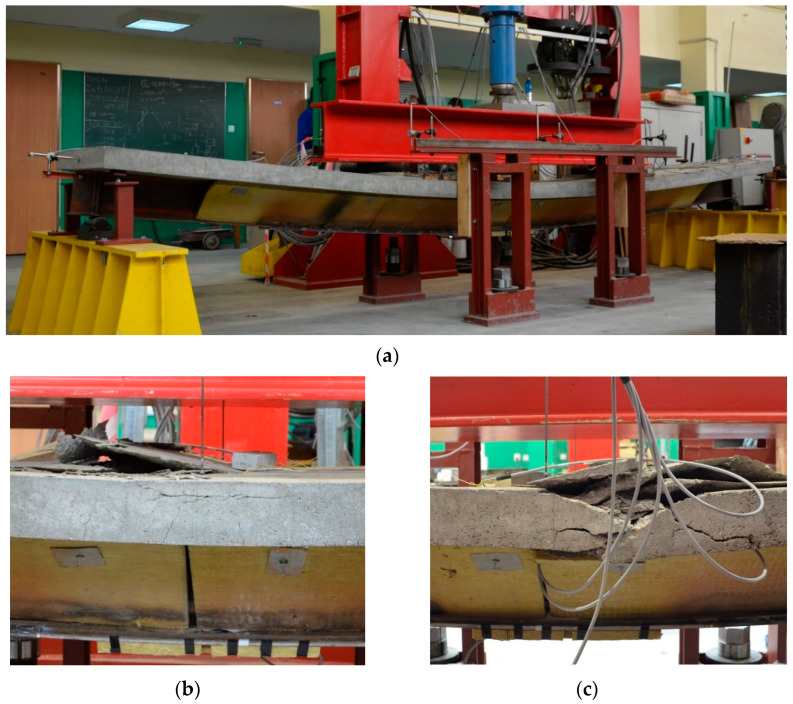
Composite beam after the test: (**a**) overview and (**b**,**c**) damages to the beam.

Once the beam had cooled completely (about 2 h after the welding was completed), the third stage began, which involved increasing the loads until the yielding and failure of the element. The setup of the hydraulic actuator was changed from force to displacement control. The test speed was adjusted so as to maintain a constant increase in concrete deformation of 1 ‰/10 min (the same speed was used to load the concrete specimens during the material tests). A clear increase in the load-bearing capacity and stiffness of the element was observed due to the operation of the strengthening plate. Once the elastic range was exceeded, a further increase in load resulted in the yielding of the bottom flange of the steel beam and a redistribution of stresses between the reinforcing and reinforced parts. Further yielding of the steel tensile part resulted in the appearance of increasing compressive deformations in the reinforced concrete slab and, finally, in the concrete exceeding its compressive strength and getting crushed (Figure 20b,c). A view of the entire beam at the end of the test is shown in Figure 20a. The whole measuring cycle consisted of multiple stages and took more than 5 h each time.

## 3. Results

The comparison of the experimental results and numerical simulations was based on two main criteria. The first, and primary, criterion is the force–displacement relationship. From this relation, it is easiest to validate the behaviour of the whole element. It is possible to clearly separate the range of the elastic work, the moment of yielding of steel and the brittle fracture of concrete. In this study, horizontal displacements were measured equally. This allowed observation of the stiffness of the steel and concrete composite. In the second step, the values of deformations were compared. This allowed verification at selected points of the structure. The strain in building structures reaches small values. Therefore, resistance strain gauges were used for measurements.

### 3.1. Displacement

A force–displacement path is used to describe the behaviour of a structural element [48]. In real-life models, force is read from a force transducer located under the hydraulic actuator. In a numerical model, it is read as the sum of the vertical reactions acting on the supports. The displacement corresponds to the deflection of the beam at the centre of the span. In the experimental study, it was read using LVDT transducers. In the numerical model, it was measured as the displacement of a selected node of the finite element mesh.

**Figure 21:**
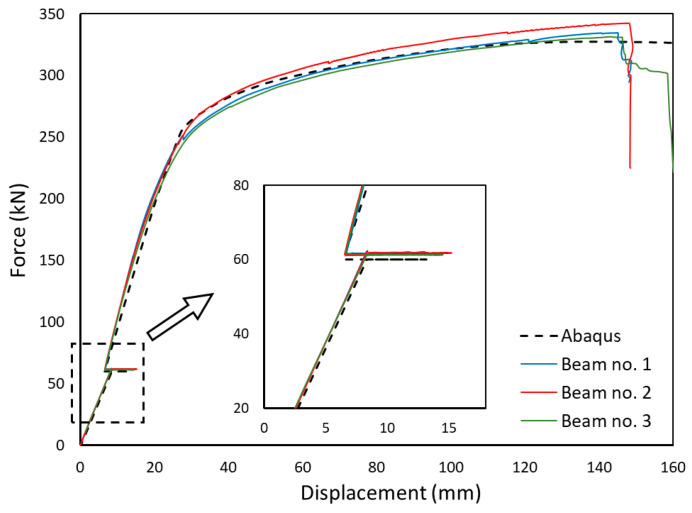
Force–displacement paths.

Figure 21 shows both the experimental (blue, red and green lines) and numerical results (black, dashed line). Given that the analysed element was partly made of concrete, it can be said that a high correlation of the results was obtained. The maximum error between the average load capacity results obtained in the tests and the numerical analysis was about 2.5%. The moment of strengthening is clearly visible on the curve. The change in the displacement value was caused by the initial thermal expansion due to welding, followed by shrinkage caused by cooling down. The displacement after welding is slightly smaller than before welding. From a limit-state point of view, this is beneficial.

Extensive displacement of the beam preceding its ultimate failure, amounting to more than 140 mm, which is 1/35 of the beam length, was observed. The failure occurred in a predictable manner in the area of constant bending moment (Figure 20b,c).

**Figure 22:**
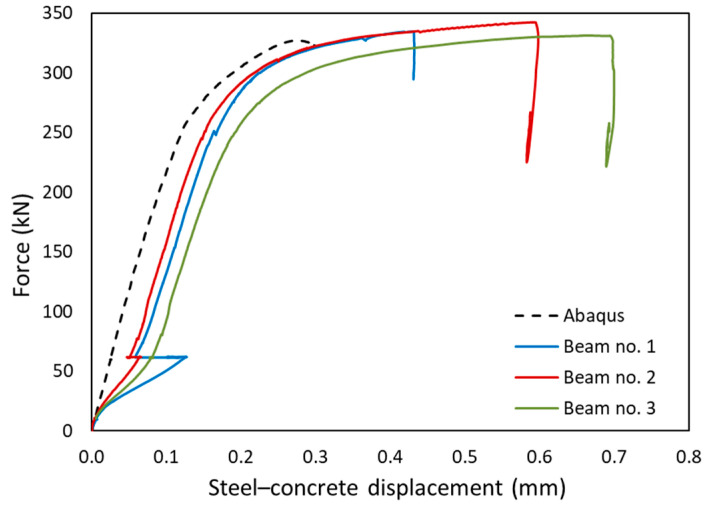
Slip at the joint plane between the I-beam and concrete slab.

Horizontal displacements occurring at the steel–concrete joint were also measured during the tests. The displacement between the reinforced concrete slab and the I-beam indicates the susceptibility of the composite in the joint plane. The shear connectors used in the tested beams are an example of a susceptible joint; thus, insignificant displacement values should occur. Figure 22 shows the obtained results. The numerical model shows slightly smaller slip values than the experimental models. The measured values are insignificant in relation to the dimensions of the model and the measuring base, and are burdened with a higher measurement error. The experimental tests also show an excessive increase in displacement during the initial loading period, which may indicate a reduction in the play near the shear connectors, as described in Section 2.6.1.

### 3.2. Strains

**Figure 23:**
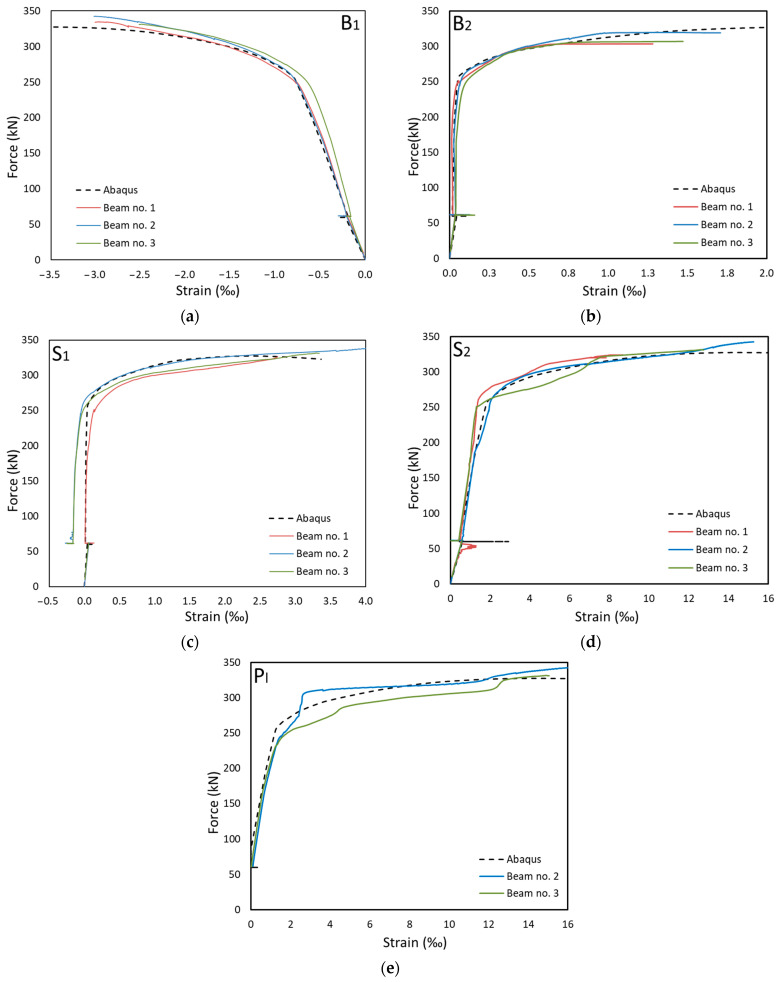
Deformation in the α-α section: (**a**) upper surface of the reinforced concrete, (**b**) bottom surface of the reinforced concrete, (**c**) upper flange of the IPE beam, (**d**) bottom flange of the IPE beam, and (**e**) strengthening plate.

The strain measurements were used to verify the numerical model. At first, the measurements taken in the α-α section located in the middle of the span were monitored. Strains were measured using five strain gauges, as shown in Figure 17. The two measurement points were located on the concrete section. Point B1 was on the top surface of the slab, while point B2 was on the bottom of the slab. Figure 23a,b present the results of the measurements taken at points B1 and B2, respectively (names of measurement points according to Figure 17). The experimental results were plotted as coloured lines, while the numerical results as a black dashed line. The obtained experimental and numerical results are characterised by a high correlation. In the case of point B1, the deformations reached 3‰, i.e., the critical working range of the concrete. Figure 23c,d show the results for the upper (S1) and bottom flanges (S2) of the I-beam, respectively. The strains on the lower surface of the strengthening plate P1 were also measured (Figure 23e). The measurements were not taken for the first of the experimental models. The strain gauge measurements are characterised by the dispersion of some results. The rapid increase in the strain values might have been caused by friction at the interface between the strengthening plate and the bottom flange of the I-beam. Overcoming the frictional forces caused a sharp increase in strain.

**Figure 24:**
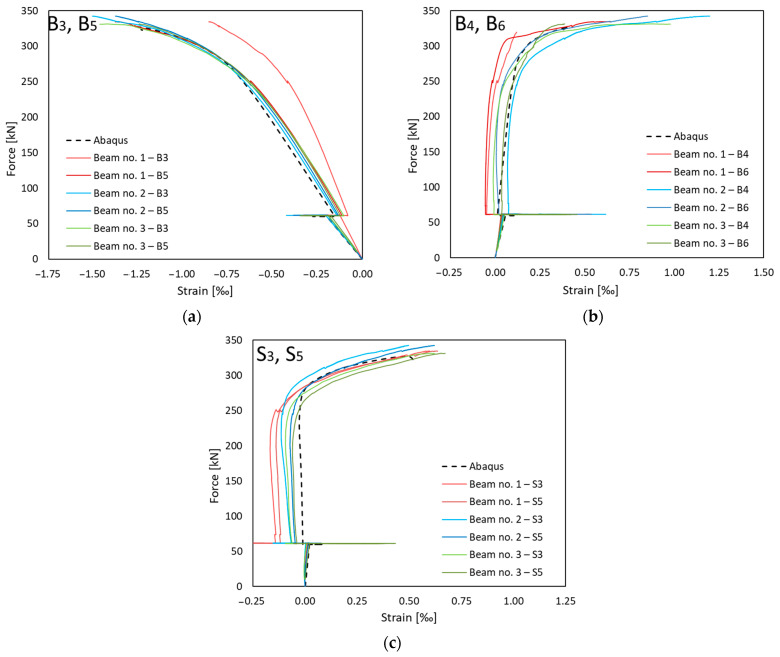
Deformations in β-β and γ-γ sections: (**a**) top surface and (**b**) bottom surface of reinforced concrete slab, and (**c**) top flange of I-beam.

In addition to the middle cross-section α-α, the strains in the cross-sections β-β and γ-γ, located outside the area of constant bending moment but still in the area of application of the strengthening plate, were determined (Figure 24). The β-β and γ-γ sections were located symmetrically at a distance of 1300 mm from the centre of the beam. Figure 24a shows the strains at the top surface of the reinforced concrete slab (B3 and B5 points according to Figure 17). Apart from one measurement point, a significant correlation of the results was obtained for both the experimental tests and the numerical model. Point B3 of beam No. 1 shows noticeably lower deformation, which may indicate an operation error. This could be due to cracks or damage to the surface of the concrete around the measuring point. Figure 24b presents the results for the bottom surface of the reinforced concrete slab at points B4 and B6. Figure 24c presents the strains recorded in the top flange of the steel beams (points S3 and S5). Unfortunately, it was not possible to record the strains in the bottom flange (S4 and S6), as these strain gauges were damaged during welding.

### 3.3. Discussion

There is a significant correlation between the results obtained through experimental tests and numerical analysis. It should be noted that the analysed structure was not homogeneous in materials, with a clear separation between ductile steel and brittle concrete. Each of these materials was modelled differently. In addition, the model takes into account welding deformations and stresses. The welding process, due to its nature and the welding method used, generated additional distortions during testing.

The similarity of the numerical and experimental results presented in Figure 21, Figure 22, Figure 23 and Figure 24 confirms the correct inclusion of these effects in the numerical model.

## 4. Summary and Conclusions

Numerical modelling is a widely used and indispensable tool in modern engineering research. FEM makes it possible to reduce time and money, and increase the number of analyses. However, numerical analysis should be verified experimentally. For the purpose of this study, a number of non-linear FEM models were prepared and optimised [50]. The numerical models were verified experimentally.

The following sections of the article describe the numerical analysis and experimental tests. To determine the materials’ properties, steel and concrete specimens were tested, and numerical, non-linear material models were prepared based on the results. The real-size specimens of the composite beam were then prepared. For both the experimental tests and numerical analysis, the research was divided into three main stages: before strengthening, during strengthening and after strengthening.

The strengthening method used in this study consisted of welding a steel plate to a member under load. The obtained results depended on a large number of factors, such as the value of stress in the weld area, the value of the linear energy of the welding arc, and the sequence and time intervals between the placement of successive welds. The safe execution of such an operation requires calculations to ensure that the limiting conditions of the structure are maintained at each stage of the strengthening process.

The final section provides a broad comparison of the results obtained from the experimental tests and the numerical analysis. The high correlation of the results, both in the linear and non-linear range of beam operation, confirmed the proper preparation and execution of the numerical analysis.

Based on the results presented above, the following conclusions can be drawn:

1. The use of the Abaqus software package allowed the precise simulation of the behaviour of the composite element, with a clear division into the brittle concrete part and the plastic steel part. Particularly, the Concrete Damage Plasticity Model was useful. The model allows for a non-linear description of the properties of concrete, independently in the analysis of compression and tension. In addition, it provides the possibility to model the stiffness degradation of concrete in the critical range and the cracking of the material.
2. Owing to the functions available in Abaqus, the process of strengthening the structure under load was modelled. From a numerical analysis point of view, this is a non-standard task, as it requires a change in the model geometry (addition of a strengthening plate) during calculation.
3. The numerical model allows the reinforced member to be tested over the full load range, including its yielding and stress redistribution between the element and the strengthening plate.
4. The properly experimentally verified numerical model allows for many subsequent numerical analyses to be conducted, without generating further research costs.
5. The research presented in this paper confirms the effectiveness of strengthening structures under load. Of course, this requires a number of technological conditions for the safe conduct of the process. However, it allows for much faster and cheaper performance of the strengthening of an existing object, without the need to remove all the equipment located on the modernised floor.
6. When a structure is strengthened under load, it is extremely important to correctly estimate welding deformations. This issue requires extended analysis in future studies.

This study analysed one of the solutions to strengthen a steel–concrete composite beam. In further research, it is planned to study an alternative solution—expansion of the concrete part by increasing its thickness. Of course, in such a case, the dead weight of the additional concrete is very important, and the initial deflection of the beam means that the thickness of the additional layer of concrete is not constant. Loads are limited only to what can be suspended to the beam from below. These will all be taken into account in future studies.

## Figures and Tables

**Table 1:** Mechanical characteristics of steel.

		*R_eh_* [MPa]	*R_el_* [MPa]	*R_m_* [MPa]	*E* [GPa]	*A* [%]
web	$\bar{x}$	368	343	482	205	26
	*s*	7.58	7.5	4.84	2.31	2.03
flange	x	328	307	460	204	31
	*s*	9.02	7.65	5.07	2.29	0.71
plate	x	271	264	383	204	36
	*s*	11.26	10.84	5.78	1.12	1.94

**Table 2:** Mechanical characteristics of concrete.

Parameter		Average Strength *f* [MPa]	Standard Deviation *s* [MPa]	Minimum Strength *f_min_* [MPa]
compression test	cubes	52.79	2.60	50.18
	cylinders	36.52	1.62	34.80
tensile test	cubes	3.41	0.22	3.14
	beams	6.43	0.43	5.99

**Table 3:** Load and corresponding stress values obtained in initial tests.

Load [kN]	Concrete		Steel	
	$\sigma_{\mathrm{concrete}}$ [MPa]	$\frac{\sigma_{\mathrm{concrete}}}{f_{\mathrm{cd}}}$	$\sigma_{\mathrm{steel}}$ [MPa]	$\frac{\sigma_{\mathrm{steel}}}{f_{y}}$
33 (2 × 10 + DL)	4.06	0.24	73.02	0.31
113 (2 × 50 + DL)	13.90	0.84	250.02	1.06

**Table 4:** Measuring points.

Measuring Point	Type	Location
P	Force transducer, HBM C6A 500 kN	α-α
f1, f2	LVDT transducer, HBM WA/200 mm	α-α
f3, f4	LVDT transducer, HBM WA/50 mm	Concentrated force (between α-α and β-β)
f5, f6	LVDT transducer, HBM WA/50 mm	Concentrated force (between α-α and γ-γ)
f7, f8	LVDT transducer, HBM WA/20 mm	Left and right ends of the beam
B1, B2	Half-bridge strain gauge measuring point	α-α
S1, S2		
B3, B4	Half-bridge strain gauge measuring point	β-β
S3, S4		
B5, B6	Half-bridge strain gauge measuring point	γ-γ
S5, S6		
P1	Half-bridge strain gauge measuring point	α-α

## Data Availability

The data presented in this study are available on request from the corresponding author.
